# When Fast Thinking Meets Bad Emotion: The Effect of Intuitive Processing Modes and Negative Moods on Moral Hypocrisy

**DOI:** 10.3390/bs15121683

**Published:** 2025-12-04

**Authors:** Binghai Sun, Pengli Yang, Liting Fan, Yuting Shao, Xin Tang

**Affiliations:** 1 Zhejiang Normal University, Jinhua 321004, China; jky18@zjnu.cn (B.S.); pleayang@zjnu.edu.cn (P.Y.); gyflt148@zjnu.edu.cn (L.F.); 2 School of Psychology, Zhejiang Normal University, Jinhua 321004, China; 3 College of Education, Zhejiang Normal University, Jinhua 321004, China

**Keywords:** moral hypocrisy, intuitive processing modes, negative moods, ego depletion, cognitive load

## Abstract

Moral hypocrisy characterized by “double standards” at the interpersonal level and “value-behavior inconsistency” at the intrapersonal level. The inconsistency in previous findings regarding the effects of intuitive processing modes on interpersonal moral hypocrisy may stem from differences in ego depletion and cognitive load. In three studies, we investigate the effects of intuitive processing modes and negative moods on moral hypocrisy. Study 1 explored the effects of these two intuitive processing modes on interpersonal hypocrisy. Study 2 further examined the combined influence of negative moods and intuitive processing modes on interpersonal hypocrisy, whereas Study 3 extended this framework from interpersonal hypocrisy to intrapersonal hypocrisy. We found that (a) under neutral mood conditions, cognitive load significantly reduced interpersonal and intrapersonal moral hypocrisy; (b) at the same time, ego depletion significantly increased interpersonal moral hypocrisy, whereas only marginally increased intrapersonal moral hypocrisy (*p* = 0.053); (c) under negative mood conditions, both intuitive processing modes increased interpersonal moral hypocrisy, but did not significantly increase intrapersonal moral hypocrisy. We further delineated the influence of distinct intuitive processing modes on moral hypocrisy.

## 1. Introduction

In everyday life, some people will try to behave morally whereas they are not actually motivated by morality (Batson et al., 1997, 1999), i.e., moral hypocrisy. Moral hypocrisy is principally characterized by two distinct types. The first, interpersonal moral hypocrisy (commonly known as “double standards”), arises when individuals judge moral behavior by applying permissive moral standards to themselves and rigorous moral standards to others (Lammers et al., 2010; Yu & Zhang, 2024). The second, intrapersonal moral hypocrisy (commonly known as “saying one thing and doing another”), is manifested when individuals’ actual moral behavior does not follow their claimed moral standards (Dong et al., 2019; Du et al., 2019). Moral hypocrisy not only damages personal moral image and interpersonal relationships (O’Connor et al., 2020; Weiss & Burgmer, 2021), but also poses threats to the generalized adherence to societal moral norms and the efficacy of intergroup cooperation (Chvaja et al., 2020). Therefore, understanding the factors influencing moral hypocrisy fosters interpersonal trust and provides guidance for moral education.

### 1.1. Intuitive Processing Modes and Moral Hypocrisy

The traditional dual process theory posits that intuitive and cognitive systems operate concurrently in moral judgment, with different moral dilemmas potentially engaging dissociable processing systems, culminating in moral decisions that reflect the results of these two systems competing with each other (Greene et al., 2001, 2008). The intuitive system, characterized by low cognitive effort and automaticity, relies on affective responses and heuristic beliefs to solve problems; in contrast, the cognitive system, requiring sustained cognitive resource allocation, employs effortful reasoning governed by logical standards to solve problems (Evans & Stanovich, 2013; Liu et al., 2025; Shi & Liu, 2019). Activation of the cognitive system typically induces suppression and override of the intuitive system, often resulting in counterintuitive results (Evans & Stanovich, 2013; Shenhav et al., 2012; Zheng et al., 2025). Previous research has pointed out that intuitive processing can influence individuals’ moral hypocrisy, but the results of existing studies are conflictive. One perspective posits that intuitive processing may effectively inhibit moral hypocrisy. Research findings indicate that individuals with intuitive thinking dominance tend to form judgments based on ethical norms when confronting ethical transgressions, thereby applying consistent moral standards to both self and others (Li et al., 2021; Valdesolo & DeSteno, 2007), at the same time doing what they say (van ’t Veer et al., 2014). In summary, these findings suggest that intuitive processing reduces moral hypocrisy across interpersonal and intrapersonal dimensions. An alternative perspective posits that individuals exhibiting intuitive processing dominance hold relatively flexible standards of judgment and can make adaptive adjustments to moral decisions, thereby amplifying interpersonal moral hypocrisy as well as intrapersonal moral hypocrisy (Du et al., 2019).

A comparative analysis of the present studies that support these two perspectives reveals divergence in their modes of intuitive processing. Ego depletion and cognitive load, as effective paradigms of suppressing cognitive processing and activating intuitive processing (Achtziger et al., 2016; Halali et al., 2014; H. Zhang et al., 2018), can influence an individual’s performance during the completion of complex cognitive tasks (Drolet & Luce, 2004; Isler & Yilmaz, 2023; Schmeichel et al., 2003). For instance, string memory tasks can impair deliberative processing and increase reliance on intuitive processing by elevating working memory load (Isler & Yilmaz, 2023). Individuals expend energy while completing the Stroop task, leading the body to conserve remaining energy by reducing effort (Baumeister et al., 2024; Dang et al., 2020). This impairs deliberative processing and consequently affects decision-making and judgment in later tasks (Capraro, 2024). Both tasks can impair an individual’s deliberative processing (or the cognitive system), thereby increasing reliance on intuitive processing. In studies supporting the hypothesis that intuitive processing reduces moral hypocrisy, the researchers induced intuitive processing by increasing participants’ cognitive load (e.g., digit-string memory task, time pressure paradigm). The researchers found that participants with high cognitive load tended to be more consistent in their evaluation of their own and others’ moral transgressions, and no instances of moral hypocrisy were detected; in contrast, participants with low cognitive load were more forgiving of themselves, and moral hypocrisy appeared to be a problem (Li et al., 2021; Valdesolo & DeSteno, 2007). In studies supporting the hypothesis that intuitive processing increases moral hypocrisy, the researchers induced intuitive processing through ego depletion (e.g., Stroop task). According to the strength resource model of self-control (Baumeister et al., 2000), individuals in an ego depletion state experience not only cognitive fatigue when confronted with complex cognitive tasks but also a reduction in self-control resources (Capraro, 2024). This resource scarcity consequently suppresses cognitive processing while amplifying reliance on intuitive processing during moral judgment (Baumeister et al., 1998; Hagger et al., 2010). Researchers employed a Stroop task to manipulate an individual’s ego-depletion state to stimulate participants’ intuitive processing, and the results demonstrated that individuals experiencing high ego-depletion exhibited significantly increased moral hypocrisy compared to those with low ego-depletion (Du et al., 2019).

The divergent effects of ego depletion and cognitive load on moral hypocrisy may arise from distinct mechanisms through which these manipulations induce intuitive processing (Maranges et al., 2017). Under cognitive load conditions, the simultaneous execution of moral decision-making and digit-string memory tasks depletes available cognitive resources, thereby constraining individuals’ capacity to defend and rationalize their moral transgressions to maintain positive self-concepts and to achieve consistency in their evaluations of the self and others, this ultimately reduces moral hypocrisy (Liu et al., 2025; Paxton et al., 2012; Valdesolo & DeSteno, 2007). The Stroop task requires sustained self-regulatory effort, which depletes self-control resources and diminishes self-regulatory capacity, consequently undermining the ability to suppress hypocritical behaviors during moral decision-making in individuals experiencing ego depletion (Baumeister & Alghamdi, 2015; Baumeister et al., 2024; Gailliot & Baumeister, 2007). Existing studies have focused on examining the effect of a specific individual intuitive processing mode on moral hypocrisy, without conducting comparative analyses of intuitive processing modes. Therefore, we aim to investigate whether differential effects emerge across various intuitive processing modes in shaping moral hypocrisy.

### 1.2. Intuitive Processing Modes, Negative Moods and Moral Hypocrisy

Negative moods refer to unpleasant feelings subjectively experienced by individuals, such as tension, anger, guilt and disgust (Gangemi et al., 2025). The social intuitionist model posits the crucial role of moods in moral judgment, conceptualizing moral decision-making as a mood-driven process (Greene et al., 2001). Previous studies have demonstrated significant differences in how affective valence influences moral judgment. Compared to individuals experiencing positive affect, those experiencing negative affect exhibit lower tolerance for unethical behaviors (Peng & Zhang, 2016; Schnall et al., 2008; Wheatley & Haidt, 2005). This difference is primarily due to the close association between moods and the intuitive versus cognitive systems: the intuitive system typically dominates over the cognitive system under positive affective states, whereas the cognitive system generally prevails over the intuitive system under negative affective states (Ye et al., 2023). Under positive affective states, individuals tend to allocate fewer cognitive resources, resulting in relatively rapid judgments (Martín & Valiña, 2023). Conversely, under negative affective states, individuals allocate greater cognitive resources, leading to more deliberate judgments and demonstrating enhanced cognitive reasoning capacity (De Vries et al., 2012; Mills et al., 2019; Xie et al., 2022). Based on the social intuitionist model (Haidt, 2001), Greene et al. (2001, 2008) proposed the dual process theory, which posits that moral hypocrisy, as a paradoxical moral phenomenon, is the result of competition between intuitive processing and cognitive processing, in other words, cognitive processing is closely related to moral hypocrisy (Li et al., 2021). When cognitive processing dominates, individuals adopt different moral standards to identical ethical transgressions occurring to themselves versus others (Warner et al., 2024). Specifically, when evaluating self-committed transgressions, individuals apply cognitive processing to rationalize behaviors through tolerant moral standards, whereas they impose stricter standards when judging identical transgressions committed by others (Harwood et al., 2011; Russell & Giner-Sorolla, 2011; Shen & Liu, 2012; Teeny et al., 2024; Wheatley & Haidt, 2005), and moral hypocrisy emerges. Emotions serve as informational inputs that guide an individual’s decision-making and judgment (Dahò, 2025) and modulate controlled cognitive processes (Helion & Ochsner, 2018). Neuroscientific evidence indicates that the amygdala is central to emotional responses, whereas the prefrontal cortex supports controlled reasoning and is implicated in cognitive dissonance (Dahò, 2025). Both the amygdala and ventromedial prefrontal cortex contribute to moral judgment. Furthermore, the ventromedial prefrontal cortex integrates inputs from regions such as the amygdala and ventral striatum (Helion & Ochsner, 2018). Negative moods typically promote the dominance of cognitive processing (Mills et al., 2019; Xie et al., 2022; Ye et al., 2023). The activation of negative emotions may readily engage cognitive reasoning in an individual’s moral judgments or decision-making. A critical question emerges when cognitive processing becomes inhibited or intuitive processing is dominant: whether negative moods can reactivate cognitive processing dominance and increase moral hypocrisy. Therefore, we aim to investigate how negative moods and intuitive processing modes jointly influence interpersonal moral hypocrisy. Furthermore, the “detached” nature between interpersonal and intrapersonal moral hypocrisy reflects shared underlying mechanisms (Sun et al., 2012), this suggests that individuals’ claims and judgments of others can be considered as declarations of moral standards, with self-judgments serving as a reflection of an individual’s behavior (Ellemers et al., 2019). Numerous studies primarily employ moral judgment task to measure interpersonal moral hypocrisy, and use task-assignment paradigm to assess intrapersonal moral hypocrisy, with the validity of both approaches having been empirically demonstrated (Dong et al., 2023; Du et al., 2019; Li et al., 2021; Lin & Miller, 2021; Weiss et al., 2018; Yu & Zhang, 2024). Accordingly, this study primarily employs these two tasks as measures of moral hypocrisy.

For this reason, we investigated the influence of intuitive processing modes and negative moods on intrapersonal moral hypocrisy, aiming to ascertain whether intuitive processing modes and negative moods exert consistent effects on moral hypocrisy at both the interpersonal and intrapersonal levels.

### 1.3. Research Overview

We test the hypotheses through three studies. Study 1 employs a digit-string memory task (cognitive load-induced intuitive processing) and a Stroop task (ego depletion-induced intuitive processing) to activate participants’ intuitive processing, initially exploring whether there are differences in the effects of intuitive processing modes on interpersonal moral hypocrisy. Study 2 investigates the interactive influence of intuitive processing modes and negative moods on interpersonal moral hypocrisy. Study 3 extends the scope from interpersonal to intrapersonal moral hypocrisy, exploring how intuitive processing and negative moods jointly shape intrapersonal moral hypocrisy. Collectively, this research proposes the following hypotheses.

**Hypothesis** **1.** *Under neutral affective conditions, cognitive load significantly reduces interpersonal moral hypocrisy, whereas ego depletion increases interpersonal moral hypocrisy.*

**Hypothesis** **2.** *Under negative affective conditions, cognitive load and ego depletion significantly increase interpersonal moral hypocrisy.*

**Hypothesis** **3.** *Under neutral affective conditions, cognitive load significantly decreases the proportion of moral hypocrites, whereas ego depletion increases the proportion of moral hypocrites.*

**Hypothesis** **4.** *Under negative affective conditions, cognitive load and ego depletion significantly increase the proportion of moral hypocrites.*

## 2. Study 1

Study 1 employed a one-way between-subjects design. The independent variable was intuitive processing modes (cognitive load group, ego depletion group, and control group). The dependent variable was the interpersonal moral hypocrisy score (calculated as the difference between self-judgment and other-judgment scores in moral evaluations). We hypothesized that cognitive load would significantly reduce interpersonal moral hypocrisy, whereas ego depletion would increase interpersonal moral hypocrisy.

### 2.1. Method

#### 2.1.1. Participants

A total of 159 non-psychology major undergraduate students were randomly recruited. We excluded 9 participants who failed cognitive load or ego depletion manipulations, resulting in a sample of 150 participants (86 females; *M*_age_ = 20.69; *SD* = 1.39). This study was approved by the University Human Research Ethics Committee, and written informed consent was obtained from all subjects. All participants received performance-based compensation upon task completion.

#### 2.1.2. Materials

**Moral judgment scenario materials.** The experimental materials were adapted from previously validated scenarios (Lammers, 2012) and modified to align with college students’ daily lives. There were eight moral transgression scenarios, categorized into “self” and “other” conditions based on the agent of action (four scenarios per condition). Each participant evaluated two moral transgression scenarios (“self” and “other”). Participants made moral judgments about the actions of different agents, with higher ratings indicating greater moral leniency. Drawing on previous research, interpersonal moral hypocrisy can be assessed using a within-subjects design (Polman & Ruttan, 2012; Yu & Zhang, 2024). Interpersonal moral hypocrisy was measured as the difference between moral judgment scores for “self” and “other” scenarios, where larger differences reflected higher levels of hypocrisy (Du et al., 2019; Lammers et al., 2010). To counterbalance order effects, half of the participants evaluated scenarios in a “self–other” sequence, while the other half used an “other–self” sequence. Additionally, participants completed an unrelated moral judgment task between the self-related and other-related scenarios, which served to decrease the likelihood of participants discerning the experimental purpose. The Cronbach’s α for the moral judgment task was 0.933 in this study (“self” condition: Cronbach’s α = 0.886; “other” condition: Cronbach’s α = 0.853).

**Cognitive load/control group manipulation materials.** In the cognitive load condition, participants were required to memorize seven complex combinations of numbers and letters (e.g., f594jn8) for 30 s. In the control condition, participants had to memorize seven identical digits or letters (e.g., 3333333, aaaaaaa) for 30 s (Valdesolo & DeSteno, 2008).

**Ego depletion group manipulation materials.** The classic Stroop paradigm (Gailliot et al., 2007; Yhan & Li, 2012) was employed to induce participants’ ego depletion state through attentional control. The experiment included four Chinese single-word meanings (red, yellow, blue, green) and four font colors (red, yellow, blue, green), comprising 16 pairings. Participants were instructed to judge the font color while ignoring the semantic meaning: pressing the S key for red, the D key for yellow, the K key for blue, and the L key for green.

**Ego depletion manipulation check (retrospective questionnaire).** Participants in the ego depletion group completed a retrospective questionnaire assessing four dimensions: perceived task difficulty, effort expenditure during the task, post-task fatigue, and perceived energy depletion. Responses were recorded on a seven-point Likert scale (1-very little, 7-very much), with higher scores indicating stronger perceived depletion (W. Fan et al., 2021). The Cronbach’s α was 0.878.

**Cognitive load/control group manipulation check (interference questionnaire).** To assess the extent to which the cognitive load task interfered with reading the moral scenarios, participants rated the following question post-task: “To what extent did the digit-string memory task interfere with your reading of the story materials?” Responses were recorded on a seven-point scale (1-very little, 7-very much), with higher scores indicating greater perceived interference (Swann et al., 2014).

#### 2.1.3. Procedure

The experimental tasks were administered via the Tclab online experimental platform with the following procedure:

First, participants completed a basic demographic information form. Second, participants were randomly assigned to one of three conditions: cognitive load group, ego depletion group, or control group. Finally, participants in the ego depletion group completed the Stroop task, filled out the retrospective questionnaire for the independent variable manipulation test, and then completed the moral judgment task; participants in the cognitive load and control groups completed the moral judgment task after the digit-string memory task, followed by the recall of strings and completed the interference questionnaire for the independent variable manipulation test; participants in the control group additionally completed the retrospective questionnaire to assess potential ego depletion effects.

### 2.2. Data Analysis

#### 2.2.1. Power Analysis

Power analysis with an effect size (*f* = 0.26) showed that 150 participants ensured sufficient power (0.80) for one-way analysis of variance (ANOVA) for three conditions.

#### 2.2.2. Assumption Checks

The Shapiro–Wilk test was conducted to assess interpersonal moral hypocrisy, showing that the data did not meet the normality assumption (*W* = 0.952, *p* < 0.001). The *F*-test maintains its robustness in controlling Type I error even when the normality assumption is violated. (Blanca et al., 2017; Bono et al., 2017). On the basis of the skewness and kurtosis for interpersonal moral hypocrisy (Skewness = 0.238 < 2, *SD* = 0.198; Kurtosis = 0.006 < 7, *SD* = 0.394), it can be concluded that the data approximate a normal distribution (Kline, 2020, p. 77; Finney & DiStefano, 2006; Zhao et al., 2024).

### 2.3. Results

#### 2.3.1. Manipulation Check

The cognitive load group (*M =* 2.900, *SD =* 1.502) scored significantly higher on interference levels than the control group (*M =* 2.140, *SD =* 1.143), *t*(98) *=* 2.848, *p =* 0.005, *d* = 0.571, 95% CI [0.230, 1.290], indicating the cognitive load manipulation was effective. The ego depletion group showed significantly higher scores than the control group across all four dimensions of perceived task difficulty, effort expenditure, fatigue, and depletion (*ps.* < 0.001), confirming the validity of the ego depletion manipulation (see Table A1).

#### 2.3.2. Effects of Different Intuitive Processing Modes on Interpersonal Moral Hypocrisy

**Figure 1:**
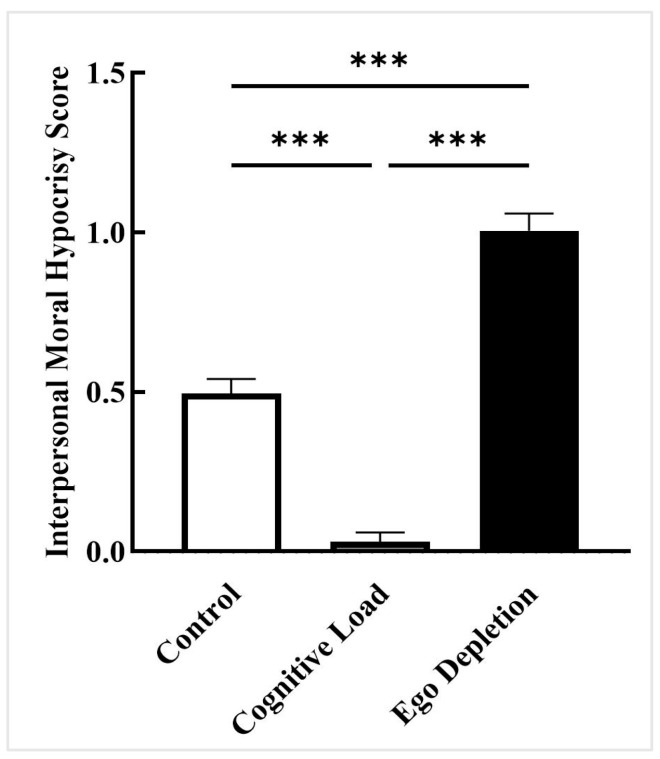
Scores of interpersonal moral hypocrisy, Study 1. Note: *** *p* < 0.001.

A one-way ANOVA was conducted with intuitive processing modes as the independent variable and interpersonal moral hypocrisy as the dependent variable. The results revealed a significant main effect of intuitive processing modes, *F*(2, 147) = 119.912, *p* < 0.001, $\eta_{p}^{2}$ = 0.620, 90% CI [0.543, 0.679] (Steiger, 2004). Multiple comparison analysis indicated that participants in the cognitive load group (*M* = 0.030, *SD* = 0.212) exhibited significantly lower interpersonal moral hypocrisy scores than those in the control group (*M* = 0.495, *SD* = 0.321, *p_bonf_* < 0.001, *d* = −1.477, 95% CI [−0.618, −0.312]). Participants in the ego depletion group (*M* = 1.005, *SD* = 0.386) demonstrated significantly higher hypocrisy scores than the control group (*p_bonf_* < 0.001, *d* = 1.620, 95% CI [0.357, 0.663]). Furthermore, the ego depletion group scored significantly higher than the cognitive load group (*p_bonf_* < 0.001, *d* = 3.096, 95% CI [0.822, 1.128]; Figure 1).

A repeated-measures ANOVA was conducted with intuitive processing modes (control group, cognitive load group, ego depletion group) and conditions (self vs. other) as independent variables and acceptability as dependent variable. The results revealed a significant main effect of intuitive processing modes, *F*(2, 147) = 4.384, *p* = 0.014, $\eta_{p}^{2}$ = 0.056. The results showed that the main effect of conditions was significant, *F*(1, 147) = 393.429, *p* < 0.001, $\eta_{p}^{2}$ = 0.728. A significant interaction effect was found between conditions and intuitive processing modes, *F*(2, 147) = 119.912, *p* < 0.001, $\eta_{p}^{2}$ = 0.620.

**Figure 2:**
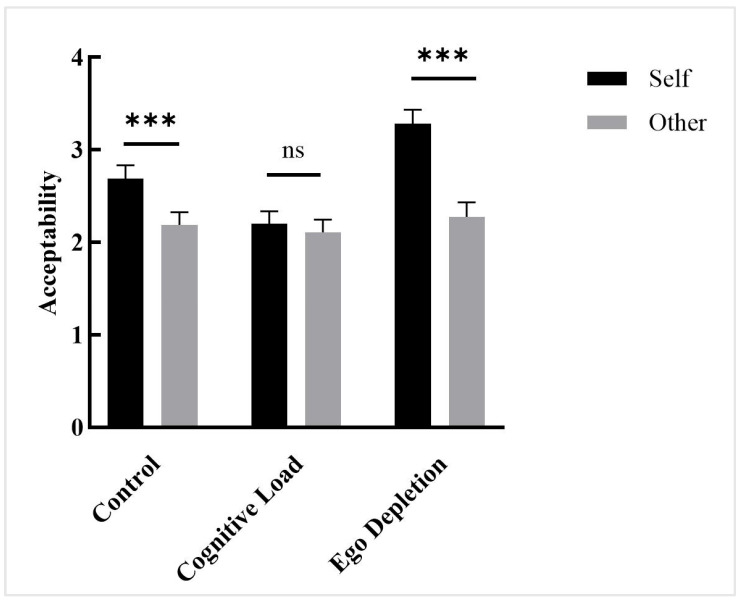
Acceptability, Study 1. Note: ns. *p* > 0.05. *** *p* < 0.001.

Further analysis indicated the following patterns (Figure 2): (a) In the control group, participants showed significantly higher acceptability of self-related scenarios than of other-related scenarios. (*p_bonf_* < 0.001, *d* = 0.487, 95% CI [0.407, 0.583]). (b) In the cognitive load group, no significant difference in acceptability was found between self-related and other-related scenarios (*p_bonf_* = 0.502, *d* = 0.030, 95% CI [−0.058, 0.118]). (c) Participants in the ego depletion group demonstrated significantly higher acceptability of self-related scenarios than of other-related scenarios (*p_bonf_* < 0.001, *d* = 0.989, 95% CI [0.917, 1.093]). These results indicate that participants in both the control and ego depletion groups demonstrated interpersonal moral hypocrisy, whereas those in the cognitive load group did not. Furthermore, the effect of ego depletion on interpersonal moral hypocrisy was more pronounced than that observed in the control group.

### 2.4. Discussion

Study 1 employed a moral judgment paradigm to examine the effects of different intuitive processing modes on interpersonal moral hypocrisy. The results confirmed Hypothesis 1: cognitive load significantly reduced interpersonal moral hypocrisy, whereas ego depletion increased it. Participants in the cognitive load group did not demonstrate interpersonal moral hypocrisy, whereas those in the ego depletion group exhibited more pronounced interpersonal moral hypocrisy. These results are partially consistent with previous findings (Du et al., 2019; Li et al., 2021). While Study 1 focused solely on how different intuitive processing modes influence interpersonal moral hypocrisy, Study 2 further investigates the combined influence of negative moods and intuitive processing modes.

## 3. Study 2

Study 2 further investigated how intuitive processing modes and negative moods jointly influence interpersonal moral hypocrisy and was based on a 3 (intuitive processing mode: ego depletion group, cognitive load group, control group) × 2 (mood type: negative moods, neutral moods) between-subjects design. The dependent variable was interpersonal moral hypocrisy (as in Study 1). We hypothesized that under neutral affective conditions, cognitive load would significantly reduce interpersonal moral hypocrisy, whereas ego depletion-induced processing would increase it; under negative affective conditions, both cognitive load and ego depletion would significantly increase interpersonal moral hypocrisy.

### 3.1. Method

#### 3.1.1. Participants

A total of 211 non-psychology major undergraduate students were randomly recruited. We excluded three participants who failed to complete experimental tasks and made patterned responses, resulting in a sample of 208 participants (133 females; *M*_age_ = 20.47; *SD* = 1.39). All participants voluntarily enrolled with no prior exposure to similar experiments.

#### 3.1.2. Material

**Mood induction materials.** The mood induction materials consisted of 20 negative affect images and 20 neutral affect images selected from the Chinese Affective Picture System (CAPS), which has been widely used for effective mood induction (Bai et al., 2005). The negative mood images included scary animals, violent scenes, and bloody scenarios, while neutral mood images included landscapes, handicrafts, and household objects.

**Positive and negative affect scale (PANAS).** The Chinese version of the PANAS consisted of 10 items assessing positive affect and 10 items assessing negative affect (Huang et al., 2003). For the current study, only the negative affect subscale was used. Participants rated their affective experiences on a 1–5 Likert scale (1-slightly, 5-extremely), with higher scores indicating stronger negative affect. The negative affect subscale demonstrated high internal consistency (Cronbach’s α = 0.854).

The moral judgment scenario materials (moral judgment task: Cronbach’s α = 0.909; “self” condition: Cronbach’s α = 0.865; “other” condition: Cronbach’s α = 0.779), the cognitive load/control group manipulation materials, the ego depletion group manipulation materials, the ego depletion manipulation check (Cronbach’s α = 0.878), and the cognitive load/control group manipulation check were identical to those used in Study 1.

#### 3.1.3. Procedure

The experimental tasks were administered via the Tclab online experimental platform with the following procedure:

First, all participants completed a basic demographic information form and took a pre-test of moods. Second, all participants were randomly assigned to one of six groups (a 3 × 2 between-subjects design). Third, participants in the neutral mood group (or negative mood group) were induced with neutral (or negative) affect by viewing landscape images (or war-related images). Fourth, participants in the ego depletion group completed the Stroop task, filled out the retrospective questionnaire for the independent variable manipulation test, and then completed the moral judgment task; participants in the cognitive load and control groups completed the moral judgment task after the digit-string memory task, followed by the recall of strings and completed the interference questionnaire for the independent variable manipulation test; participants in the control group additionally completed the retrospective questionnaire to assess potential ego depletion effects. Finally, all participants took a post-test of moods.

### 3.2. Data Analysis

#### 3.2.1. Power Analysis

Power analysis with a medium effect size (*f* = 0.25) showed that 208 participants ensured sufficient power (0.80) for repeated-measures ANOVA.

Power analysis with a medium effect size (*f* = 0.25) showed that 208 participants ensured sufficient power (0.80) for 3 × 2 ANCOVA.

#### 3.2.2. Assumption Checks

The Shapiro–Wilk test was conducted to assess negative moods, showing that the data did not meet the normality assumption (*W* = 0.943, *p* < 0.001). On the basis of the skewness and kurtosis for negative moods (Skewness = 0.761 < 2, *SD* = 0.169, Kurtosis = 0.118 < 7, *SD* = 0.336), it can be concluded that the data approximate a normal distribution (Blanca et al., 2023; Kline, 2020, p. 77; Finney & DiStefano, 2006; Zhao et al., 2024).

The Shapiro–Wilk test was conducted to assess interpersonal moral hypocrisy, showing that the data did not meet the normality assumption (*W* = 0.967, *p* < 0.001). On the basis of the skewness and kurtosis for interpersonal moral hypocrisy (Skewness = 0.109 < 2, *SD* = 0.169, Kurtosis = −0.419 < 7, *SD* = 0.336), it can be concluded that the data approximate a normal distribution (Kline, 2020, p. 77; Finney & DiStefano, 2006; Zhao et al., 2024).

### 3.3. Results

#### 3.3.1. Manipulation Check

The cognitive load group (*M =* 3.174, *SD =* 1.552) scored significantly higher on interference levels than the control group (*M =* 2.405, *SD =* 1.458), *t*(136) *=* 2.995, *p =* 0.003, *d* = 0.510, 95% CI [0.261, 1.275], indicating the cognitive load manipulation was effective. The ego depletion group showed significantly higher scores than the control group across all four dimensions of perceived task difficulty, effort expenditure, fatigue, and depletion (*ps.* < 0.010), confirming the validity of the ego depletion manipulation (see Table A2).

A repeated-measures ANOVA was conducted with mood type (negative mood vs. neutral mood) and time point (pre-test vs. post-test) as independent variables, and participants’ negative mood scores as the dependent variable. The results revealed that under negative mood conditions, post-test scores (*M* = 20.519, *SD* = 6.015) were significantly higher than pre-test scores (*M* = 9.567, *SD* = 6.849), *p* < 0.001, *d* = 1.729, 95% CI [10.080, 11.824], confirming the effectiveness of negative mood induction. However, a significant difference was found between the pre-test scores in the negative mood condition (*M =* 9.567, *SD* = 6.849) and those in the neutral mood condition (*M* = 7.375, *SD* = 5.543), *p* = 0.012, *d* = 0.346, 95% CI [0.489, 3.896]. Consequently, pre-test scores were included as covariates in subsequent analyses (see Table A3 and Table A4).

#### 3.3.2. Effects of Intuitive Processing Modes and Negative Moods on Interpersonal Moral Hypocrisy

An ANCOVA was conducted with mood type (negative vs. neutral) and intuitive processing modes (control group, cognitive load group, ego depletion group) as independent variables, pre-test mood scores as a covariate, and interpersonal moral hypocrisy scores as dependent variable. The results showed a significant main effect of mood type, *F*(1, 201) = 176.950, *p* < 0.001, $\eta_{p}^{2}$ = 0.468, 90% CI [0.389, 0.537]. Participants in the negative mood group (*M* = 1.212, *SD* = 0.429) exhibited significantly higher interpersonal moral hypocrisy scores than those in the neutral mood group (*M* = 0.469, *SD* = 0.514, *d* = 1.873, 95% CI [0.622, 0.838]). The results showed a significant main effect of intuitive processing modes, *F*(2, 201) = 30.370, *p* < 0.001, $\eta_{p}^{2}$ = 0.232, 90% CI [0.150, 0.310]. Further analysis revealed that the ego depletion group (*M* = 1.121, *SD* = 0.556) scored significantly higher than both the control group (*M* = 0.764, *SD* = 0.402; *p* < 0.001, *d* = 0.892, 95% CI [0.188, 0.508]) and the cognitive load group (*M* = 0.630, *SD* = 0.703; *p* < 0.001, *d* = 1.290, 95% CI [0.343, 0.662]). No significant difference was found between the cognitive load group and the control group (*p* = 0.063, *d* = 0.398, 95% CI [−0.006, 0.316]).

**Figure 3:**
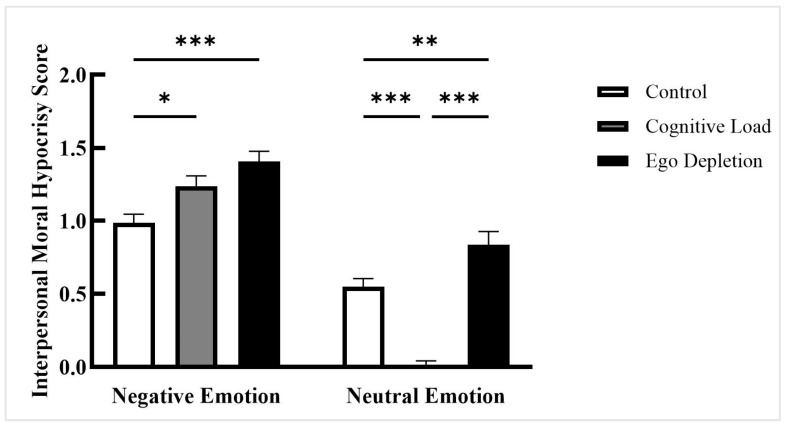
Scores of interpersonal moral hypocrisy, Study 2. Note: * *p* < 0.05. ** *p* < 0.01. *** *p* < 0.001.

A significant interaction effect was found between affect type and intuitive processing modes, *F*(2, 201) = 20.688, *p* < 0.001, $\eta_{p}^{2}$ = 0.171, 90% CI [0.095, 0.245]. Further analysis revealed the following patterns (Figure 3): (a) Under neutral mood conditions, the cognitive load group (*M* = 0.007, *SD* = 0.209) exhibited significantly lower interpersonal moral hypocrisy scores than both the control group (*M* = 0.550, *SD* = 0.331; *p_bonf_* < 0.001, *d* = −1.421, 95% CI [0.369, 0.739]) and the ego depletion group (*M* = 0.836, *SD* = 0.539; *p_bonf_* < 0.001, *d* = −2.140, 95% CI [−1.061, −0.607]); the ego depletion group scored significantly higher than the control group (*p_bonf_* = 0.009, *d* = 0.721, 95% CI [0.056, 0.506]). (b) Under negative mood conditions, the cognitive load group (*M* = 1.236, *SD* = 0.424) demonstrated significantly higher hypocrisy scores than the control group (*M* = 0.985, *SD* = 0.348; *p_bonf_* = 0.031, *d* = 0.625, 95% CI [0.017, 0.470]); the ego depletion group (*M* = 1.407, *SD* = 0.412) also scored significantly higher than the control group (*p_bonf_* < 0.001, *d* = 1.064, 95% CI [0.188, 0.642]); no significant difference was observed between the cognitive load and ego depletion groups (*p_bonf_* = 0.203, *d* = 0.439, 95% CI [−0.396, 0.054]).

A repeated-measures ANOVA was conducted with mood types (negative vs. neutral), intuitive processing modes (control group, cognitive load group, ego depletion group), and conditions (self vs. other) as independent variables, pre-test mood scores as a covariate, and acceptability as dependent variable. The results revealed a significant main effect of intuitive processing modes, *F*(2, 201) = 5.430, *p* = 0.005, $\eta_{p}^{2}$ = 0.051. The results showed that the main effect of mood types was significant, *F*(1, 201) = 6.188, *p* = 0.014, $\eta_{p}^{2}$ = 0.030. The results revealed a significant main effect of conditions, *F*(1, 201) = 287.965, *p* < 0.001, $\eta_{p}^{2}$ = 0.589. The results showed that the interaction between mood types and intuitive processing modes was non-significant, *F*(2, 201) = 2.359, *p* = 0.097, $\eta_{p}^{2}$ = 0.023. A significant interaction effect was found between conditions and intuitive processing modes, *F*(2, 201) = 30.370, *p* < 0.001, $\eta_{p}^{2}$ = 0.232. The results showed that the interaction between conditions and mood types was significant, *F*(1, 201) = 176.950, *p* < 0.001, $\eta_{p}^{2}$ = 0.468. The three-way interaction of conditions, mood types, and intuitive processing modes was also significant, *F*(2, 201) = 20.688, *p* < 0.001, $\eta_{p}^{2}$ = 0.171.

**Figure 4:**
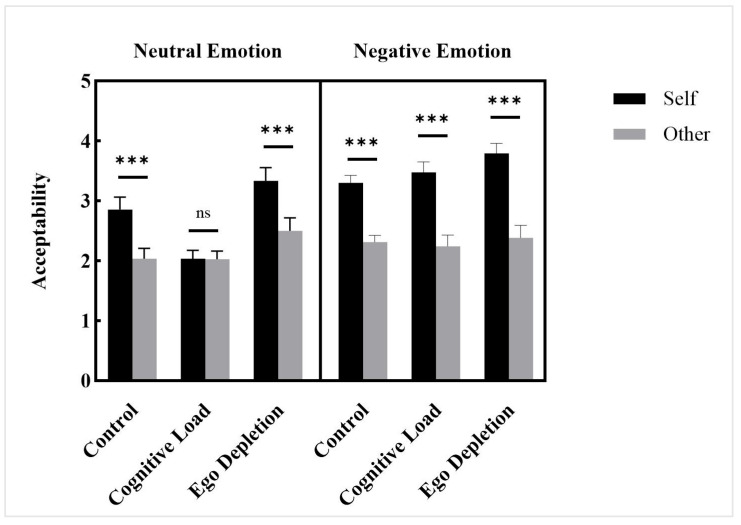
Acceptability, Study 2. Note: ns. *p* > 0.05. *** *p* < 0.001.

Further analysis revealed the following patterns (Figure 4): (a) Under the neutral emotion condition, participants in the control group demonstrated significantly higher acceptability of self-related scenarios than other-related scenarios (*p_bonf_* < 0.001, *d* = 0.545, 95% CI [0.366, 0.760]). Participants in the cognitive load group showed no significant difference in acceptability between self-related and other-related scenarios (*p_bonf_* = 1.000, *d* = 0.009, 95% CI [−0.189, 0.208]). Participants in the ego depletion group exhibited significantly higher acceptability of self-related scenarios compared to other-related scenarios (*p_bonf_* < 0.001, *d* = 0.818, 95% CI [0.647, 1.040]). (b) Under the negative emotion condition, participants in the control group demonstrated significantly higher acceptability of self-related scenarios than other-related scenarios (*p_bonf_* < 0.001, *d* = 0.952, 95% CI [0.784, 1.181]). Participants in the cognitive load group showed significantly higher acceptability of self-related scenarios than other-related scenarios (*p_bonf_* < 0.001, *d* = 1.188, 95% CI [1.029, 1.422]). Participants in the ego depletion group exhibited even more pronounced acceptability of self-related scenarios compared to other-related scenarios (*p_bonf_* < 0.001, *d* = 1.354, 95% CI [1.201, 1.594]).

Under the neutral emotion condition, both the control and ego depletion groups demonstrated interpersonal moral hypocrisy, whereas the cognitive load group did not. These results are consistent with the findings from Study 1. Under the negative emotion condition, interpersonal moral hypocrisy was observed in all groups (control, cognitive load, and ego depletion), with notably stronger effects across conditions.

### 3.4. Discussion

Study 2 introduced negative moods to further explore its role in how intuitive processing modes influence interpersonal moral hypocrisy based on Study 1. The results showed that in the neutral mood condition, cognitive load significantly reduced interpersonal moral hypocrisy, whereas ego depletion increased it. These findings supported Hypothesis 1 and aligned with the results of Study 1, reaffirming the distinct effects of the two intuitive processing modes. In the negative mood condition, both the cognitive load group and the ego depletion group exhibited significantly higher levels of interpersonal moral hypocrisy than the control group, while no significant difference was found between the cognitive load and ego depletion groups. This indicates that negative moods exert a stronger influence on interpersonal moral hypocrisy. Study 3 will investigate the combined effects of negative moods and intuitive processing modes on intrapersonal moral hypocrisy.

## 4. Study 3

Study 3 extends the investigation from interpersonal to intrapersonal moral hypocrisy, examining the effects of intuitive processing modes and negative moods on intrapersonal moral hypocrisy. This study was based on a 3 (intuitive processing mode: ego depletion group, cognitive load group, control group) × 2 (mood type: negative moods, neutral moods) between-subjects design. The dependent variable was the proportion of moral hypocrites (task choice consistency). We hypothesized that: under neutral affective conditions, cognitive load would significantly reduce the proportion of moral hypocrites, whereas ego depletion would significantly increase it; under negative affective conditions, both cognitive load and ego depletion would significantly increase the proportion of moral hypocrites.

### 4.1. Method

#### 4.1.1. Participants

A total of 220 non-psychology major undergraduate students were randomly recruited. We excluded nine participants who failed to complete experimental tasks and made patterned responses, resulting in a sample of 211 participants (133 females; *M*_age_ = 19.71; *SD* = 1.03).

#### 4.1.2. Material

**Task assignment paradigm (TAP).** Adapted from the task-assignment paradigm proposed by Batson et al. (1999). Participants were first informed that they would complete two tasks with another participant (fictitious), with each assigned to one task. Next, participants entered the role assignment session, where they were told the system had randomly selected them as the task allocator. They read the instructions: “Please choose a fair allocation method. The receiver will unconditionally accept your assigned task. Your identities will remain confidential to each other.” Participants then chose between two allocation methods (direct allocation or random allocation). Those selecting direct allocation directly wrote down their preferred task number, while those choosing random allocation pressed a key on the computer to initiate a random draw. After scrolling, the screen displayed the “randomly draw” task (“1” for a picture-matching game, “2” for math problem-solving). The system was preprogrammed to display a fixed outcome: “Your assigned task is math problem-solving.” Finally, participants reported their final chosen task number. Both the picture-matching game and math problem-solving were fictitious tasks requiring no actual completion. Intrapersonal moral hypocrisy was measured by the consistency between the system-assigned task and the participant’s final chosen task.

The cognitive load/control group manipulation materials, the ego depletion group manipulation materials, the ego depletion manipulation check (Cronbach’s α = 0.898), and the cognitive load/control group manipulation check were identical to those used in Study 1.

Mood induction materials, the Chinese version of the PANAS (Cronbach’s α = 0.734) were identical to those used in Study 2.

#### 4.1.3. Procedure

The experimental tasks were administered via the Tclab online experimental platform with the following procedure:

First, all participants completed a basic demographic information form and took a pre-test of moods. Second, all participants were randomly assigned to one of six groups (a 3 × 2 between-subjects design). Third, participants in the neutral mood group (or negative mood group) were induced with neutral (or negative) affect by viewing landscape images (or war-related images). Fourth, participants in the ego depletion group completed the Stroop task, filled out the retrospective questionnaire for the independent variable manipulation test, and then completed the TAP; participants in the cognitive load and control groups completed the TAP after the digit-string memory task, followed by the recall of strings and completed the interference questionnaire for the independent variable manipulation test; participants in the control group additionally completed the retrospective questionnaire to assess potential ego depletion effects. Finally, all participants took a post-test of moods.

### 4.2. Data Analysis

#### 4.2.1. Power Analysis

Power analysis with a medium effect size (*f* = 0.25) showed that 211 participants ensured sufficient power (0.80) for repeated-measures ANOVA.

#### 4.2.2. Assumption Checks

The Shapiro–Wilk test was conducted to assess negative moods, showing that the data did not meet the normality assumption (*W* = 0.975, *p* < 0.001). On the basis of the skewness and kurtosis for negative moods (Skewness = 0.382 < 2, *SD* = 0.167, Kurtosis = 0.052 < 7, *SD* = 0.333), it can be concluded that the data approximate a normal distribution (Blanca et al., 2023; Kline, 2020, p. 77; Finney & DiStefano, 2006; Zhao et al., 2024).

### 4.3. Results

#### 4.3.1. Manipulation Check

The cognitive load group (*M =* 3.243, *SD =* 1.209) scored significantly higher on interference levels than the control group (*M =* 2.014, *SD =* 1.014), *t*(138) *=* 6.513, *p <* 0.001, *d* = 1.102, 95% CI [0.746, 1.457], indicating the cognitive load manipulation was effective. The ego depletion group showed significantly higher scores than the control group across all four dimensions of perceived task difficulty, effort expenditure, fatigue, and depletion (*ps.* < 0.001), confirming the validity of the ego depletion manipulation (see Table A5).

A repeated-measures ANOVA was conducted with mood type (negative mood vs. neutral mood) and time point (pre-test vs. post-test) as independent variables, and participants’ negative mood scores as the dependent variable. The results showed no significant difference between participants’ pre-test scores in the negative mood condition (*M* = 7.170, *SD* = 2.535) and those in the neutral mood condition (*M* = 7.848, *SD* = 2.752), *p* = 0.064, *d* = −0.182, 95% CI [−1.396, 0.040]; under negative mood conditions, participants’ post-test scores (*M* = 12.972, *SD* = 4.086) were significantly higher than their pre-test scores (*M* = 7.170, *SD* = 2.535), *p* < 0.001, *d* = 1.558, 95% CI [4.974, 6.630], confirming the effectiveness of negative mood induction (see Table A6 and Table A7).

#### 4.3.2. Effects of Intuitive Processing Modes and Negative Moods on Intrapersonal Moral Hypocrisy

After excluding 54 participants who chose direct task allocation, a chi-squared analysis was performed on the task choice consistency of the remaining participants who selected random allocation (*N* = 157). Power analysis with a medium effect size (φ = 0.30) showed that 157 participants ensured sufficient power (0.80) for $\chi^{2}$ test. The results showed a significant effect of mood type on intrapersonal moral hypocrisy (see Table 1). The proportion of moral hypocrites was significantly higher under negative mood conditions than under neutral mood conditions, $\chi^{2}$(1, *N* = 157) = 22.466, *p* < 0.001, φ = 0.378. Additionally, different intuitive processing modes had a significant effect on intrapersonal moral hypocrisy, $\chi^{2}$(2, *N* = 157) = 12.952, *p* = 0.002, $\varphi_{c}$ = 0.287. Further analyses revealed that significant differences were observed across intuitive processing modes under neutral mood conditions ($\chi^{2}$(2, *N* = 80) = 22.689, *p* < 0.001, $\varphi_{c}$ = 0.533), the proportion of moral hypocrites in the cognitive load group was significantly lower compared to that in the control group ($\chi^{2}$(1, *N* = 53) = 9.049, *p* = 0.005, $\varphi_{c}$ = 0.413), the proportion of moral hypocrites in the ego depletion group was marginally higher than in the control group ($\chi^{2}$(1, *N* = 52) = 3.729, *p* = 0.053, $\varphi_{c}$ = 0.268). No significant differences were found across intuitive processing modes under negative mood conditions, $\chi^{2}$(2, *N* = 77) = 0.095, *p* = 0.953.

### 4.4. Discussion

Study 3 extended the investigation from interpersonal to intrapersonal moral hypocrisy, exploring the effects of intuitive processing modes and negative moods on intrapersonal moral hypocrisy. The results showed that under neutral affective conditions, the proportion of moral hypocrites was significantly reduced in the cognitive load group compared to the control group, while the ego depletion group showed a slightly higher proportion (*p* = 0.053). These findings did not fully support Hypothesis 3 and diverged partially from the results of Studies 1 and 2. Under negative affective conditions, no significant differences in the proportion of moral hypocrites were observed across intuitive processing modes, failing to support Hypothesis 4.

## 5. General Discussion

Through three studies, we investigated the effects of intuitive processing modes and negative moods on moral hypocrisy. Study 1 examined the impact of different intuitive processing modes (cognitive load vs. ego depletion) on interpersonal moral hypocrisy. Study 2 further explored how intuitive processing modes and negative affect jointly influence interpersonal moral hypocrisy. Study 3 expanded the scope of moral hypocrisy from the interpersonal to the intrapersonal level by exploring the effects of intuitive processing modes and negative moods on intrapersonal moral hypocrisy. The results demonstrated that: under neutral affective conditions, cognitive load significantly reduced interpersonal moral hypocrisy, while ego depletion significantly increased it; cognitive load significantly decreased the proportion of moral hypocrites, but ego depletion resulted in a non-significant increase. Under negative affective conditions, both cognitive load and ego depletion increase interpersonal moral hypocrisy; however, neither processing mode significantly increased the proportion of moral hypocrites.

### 5.1. Effects of Intuitive Processing Modes on Moral Hypocrisy

The findings of this study indicate that cognitive load-induced intuitive processing reduces interpersonal moral hypocrisy. When evaluating their unethical behaviors, individuals engage in conscious cognitive processing to rationalize such actions and preserve their moral self-image—a process requiring greater cognitive resources (Li et al., 2021; Martin et al., 2021). In contrast, when judging others’ moral transgressions, the evaluation relies on intuitive processing driven by an “innate aversion”, as it does not necessitate safeguarding others’ interests or reputations (L. Fan et al., 2025), thereby demanding fewer cognitive resources (Valdesolo & DeSteno, 2008). Furthermore, according to the cognitive resource limitation theory, all cognitive activities consume cognitive resources, and the total capacity of cognitive resources is limited, so it is difficult for individuals to simultaneously perform multiple tasks or process diverse complex information (Kirschner et al., 2018; van Merriënboer & Sweller, 2005). As the cognitive resources allocated to one task increase, those available for another task correspondingly decrease (Isler & Yilmaz, 2023). In brief, when cognitive load tasks occupy a portion of an individual’s cognitive resources, the remaining resources allocated to other tasks diminish, thereby inhibiting deliberate cognitive processing (Liu et al., 2025). This forces individuals to rely on intuitive processing when making moral judgments (Mieth et al., 2021; Zheng et al., 2025). Under intuitive processing dominance, individuals tend to base their judgments of moral transgressions on ethical principles, applying consistent moral standards to both themselves and others, which reduces interpersonal moral hypocrisy. This indicates that individuals with predominant intuitive processing lack sufficient cognitive resources to preserve moral self-image, rendering “double-standard” strategies unattainable.

The findings of this study also indicate that ego depletion-induced intuitive processing increases interpersonal moral hypocrisy. According to the traditional dual process theory, individuals in an ego depletion-induced intuitive processing state should exhibit no significant differences in moral judgments toward themselves and others. However, these results appear inconsistent with the traditional dual process theory explanation of moral hypocrisy, primarily because people desire to be perceived as moral, and maintaining a positive moral self-image requires willpower (self-control) to balance self-interest and altruism when demonstrating moral behavior (L. Fan et al., 2025; Valdesolo & DeSteno, 2007). Self-control, termed the “moral muscle”, refers to the ability to overcome self-interest and act morally (Baumeister & Exline, 1999; Weiss et al., 2021), which is crucial for maintaining moral self-image and pursuing moral reputation (Du et al., 2019). Although ego depletion inhibits participants’ cognitive processing, making them more prone to rely on intuitive processing (Capraro, 2024; Achtziger et al., 2016; Halali et al., 2014), the self-control resource model (Baumeister et al., 1998, 2000) posits that after ego depletion, self-control resources are depleted and individuals neglect altruistic concerns and focus more on self-interest. Whether to conserve remaining energy or due to insufficient motivation, individuals tend to reduce effort in subsequent tasks. This reduction represents a decline in self-control effort resulting from ego depletion (Baumeister et al., 2024). That is to say, individuals with depleted self-control resources cannot prevent suppression of the “inherently hypocritical human nature”, leading to the emergence of interpersonal moral hypocrisy. Furthermore, the separation between moral judgment standards for oneself versus others in interpersonal moral hypocrisy is fundamentally equivalent to the disconnect between professed moral standards and actual behavior in intrapersonal moral hypocrisy. The moral judgment standard applied to others inherently reflects another manifestation of one’s internalized moral standards (Ellemers et al., 2019; Sun et al., 2012). Specifically, in the relationship between cognitive load and intrapersonal moral hypocrisy, after committing an ethical transgression, individuals lack sufficient cognitive resources to rationalize their unethical behavior (i.e., self-deception). Consequently, they can only compare their actions against their claimed moral principles, reducing the “value–behavior inconsistency” characteristic of moral hypocrisy (Batson et al., 1997; Greene et al., 2001, 2008).

The differential effects of intuitive processing modes on moral hypocrisy arise primarily because not all such modes inhibit cognitive processing solely through cognitive resource depletion (Maranges et al., 2017). Unlike cognitive load-induced intuitive processing, ego depletion-induced intuitive processing decreases individuals’ self-control resources. Consequently, ego depletion makes it difficult to suppress the emergence of moral hypocrisy. In contrast, cognitive load merely increases the consumption of cognitive resources without impacting self-control resources, thereby reducing individuals’ moral hypocrisy.

Both cognitive load and ego depletion suppress deliberative processing while promoting greater reliance on intuitive processing (Baumeister et al., 2024; Isler & Yilmaz, 2023). Research suggests that individuals’ moral decision-making and judgment depend on distinct types of intuitive processing (Bago & De Neys, 2019; Bago et al., 2021). Intuitive processing can be characterized as either heuristic-based or logic-based (Capraro, 2024; De Neys, 2023). This implies that cognitive load-induced intuitive processing may possess a “heuristic” advantage that reduces moral hypocrisy, whereas ego depletion-induced intuitive processing may demonstrate a “logical” advantage that leads to promoting moral hypocrisy.

### 5.2. Effects of Intuitive Processing Modes and Negative Moods on Moral Hypocrisy

Study 2 further investigated the combined effects of intuitive processing modes and negative moods on interpersonal moral hypocrisy based on Study 1, while Study 3 extended the exploration from interpersonal to intrapersonal moral hypocrisy. The findings demonstrate that both cognitive load-induced and ego depletion-induced intuitive processing increased interpersonal moral hypocrisy in the negative mood condition, reflecting that negative moods exert a stronger influence on interpersonal moral hypocrisy. Previous research indicates that moods influence moral judgment, with negative moods leading individuals to adopt stricter moral standards and exhibit lower tolerance for moral transgressions (Peng & Zhang, 2016; Schnall et al., 2008; Wheatley & Haidt, 2005). Meanwhile, some studies have found that affective valence is related to cognitive processing. For example, compared to positive affective states, individuals in negative affective states demonstrate enhanced performance in cognitive processing due to accelerated consolidation of working memory (Xie et al., 2022). Individuals in negative affective states perform better in cognitive reasoning tasks compared to those in positive affective states (Mills et al., 2019). Individuals experiencing positive moods tend to focus more on superficial and extraneous information, which hinders reasoning and problem-solving. In contrast, those in negative moods are more likely to concentrate on local, detailed information and engage in analytical thinking (Ye et al., 2023). This suggests that different types of intuition may have distinct characteristics: positive emotions might align with a “heuristic” processing style, whereas negative emotions could be associated with a “logical” processing style (Capraro, 2024). This suggests that individuals in positive affective states rely on heuristic experience for rapid decision-making, whereas those in negative affective states prioritize rule-based logic to maximize benefits. In the competition between cognitive and intuitive processing, negative mood may enable cognitive processing to gain dominance, pulling individuals back from an induced intuitive state into a cognitive processing state. Cognitive processing plays a critical role in self-justification and rationalization of individuals’ own transgressions (Paxton et al., 2012). Individuals actively seek excuses to defend their transgressions to preserve a positive self-concept, while exhibiting automatic negative reactions toward others’ transgressions, leading to more interpersonal moral hypocrisy.

Under negative emotional conditions, both cognitive load and ego depletion were found to increase interpersonal moral hypocrisy. However, this result cannot be fully explained by traditional dual-process theory. Integrating recent advances in dual-process theory, an alternative explanation may be proposed. Based on the core assumptions of dual process 2.0 models, when negative moods and cognitive load (or ego depletion) are simultaneously activated, the comparable strength of these two intuitive processes may lead to competition between them (De Neys, 2023; De Neys & Pennycook, 2019). Once the intensity of this conflict reaches a certain threshold, it can trigger deliberation to generate a final response (Capraro, 2024), thereby enabling individuals to rationalize their own moral transgressions and ultimately increasing interpersonal moral hypocrisy.

From another perspective, different categories of intuition may vary in their directional tendencies, which can influence the competitive dynamics between distinct intuitive processes (Bago & De Neys, 2019; Bago et al., 2021; De Neys, 2023). In terms of types of intuition, there may exist intuitive processing characterized by heuristic and logical features (Capraro, 2024; De Neys, 2023). This implies that cognitive load may exhibit a “heuristic” character, while negative moods and ego depletion might possess a “logical” character. Negative moods could compete with cognitive load, and due to their higher activation intensity, negative moods may exert a stronger influence on interpersonal moral hypocrisy as the more dominant intuitive process. Meanwhile, since both negative moods and ego depletion likely share a “logical” advantage, they may not compete with each other (Bago & De Neys, 2019; Capraro, 2024; De Neys & Pennycook, 2019). Given their aligned directional tendencies, they may jointly amplify interpersonal moral hypocrisy.

### 5.3. Differences Between Interpersonal and Intrapersonal Moral Hypocrisy

A comparison of the results from Studies 2 and 3 revealed that under negative mood conditions, both the cognitive load group and ego depletion group exhibited significantly higher interpersonal moral hypocrisy scores than the control group, whereas the proportion of moral hypocrites in the cognitive load and ego depletion groups showed no significant differences compared to the control group.

The results of these two studies are inconsistent, and one contributing factor lies in the differences between the tasks used to measure moral hypocrisy. Interpersonal moral hypocrisy was assessed using a moral judgment task, where participants evaluated their acceptability of moral transgressions committed by different protagonists (“self” and “others”) (Weiss et al., 2018). If participants showed higher acceptability of their own moral transgressions than those of others, it indicated the presence of interpersonal moral hypocrisy. At the same time, the greater the disparity in acceptability between “self” and “others”, the higher the degree of interpersonal moral hypocrisy (Dong et al., 2023; Lammers, 2012). Intrapersonal moral hypocrisy was measured using the task assignment paradigm. In this paradigm, participants were required to allocate a simple and enjoyable task versus a complex and tedious task. If participants chose to assign the system-assigned complex and tedious task to others while allocating the simple and enjoyable task to themselves, it indicated the presence of intrapersonal moral hypocrisy (Du et al., 2019). In summary, the moral judgment task not only identifies the presence of interpersonal moral hypocrisy but also distinguishes its degree. In contrast, the task assignment paradigm merely detects the existence of intrapersonal moral hypocrisy without quantifying its degree. This limitation results in constrained statistical approaches for analyzing data from the task assignment paradigm—typically limited to chi-squared tests for count data—thereby compromising both data utility and result robustness.

On the other hand, the inconsistency between the results of the two studies stems from differences in the diagnostic difficulty of interpersonal versus intrapersonal moral hypocrisy. Interpersonal moral hypocrisy is assessed by whether individuals apply the same moral standards to judge their own and others’ moral transgressions, and intrapersonal moral hypocrisy is evaluated based on the consistency between professed moral standards and actual behaviors (Dong et al., 2022, 2023). More importantly, identifying interpersonal moral hypocrisy is relatively more challenging, whereas detecting intrapersonal moral hypocrisy is simpler (Wu & Gao, 2013). Specifically, the difficulty in identifying interpersonal moral hypocrisy arises because it requires indirectly inferring the presence of a double moral standard by comparing individuals’ judgments of their own versus others’ moral transgressions. In contrast, detecting intrapersonal moral hypocrisy is simpler because it merely requires comparing the moral standards individuals previously professed with their actual behaviors. Individuals always seek behavioral outcomes that align with self-interest without incurring self-blame. When realizing this goal is unattainable, they engage in self-serving rationalization motivated by self-interest, a process requiring the involvement of cognitive processing (Lin & Miller, 2021; Paxton et al., 2012). Concurrently, negative moods can also enhance cognitive processing (Xie et al., 2022; Ye et al., 2023). When deliberation intervenes in the competitive interaction between negative moods and cognitive load (or ego depletion) (De Neys, 2023), individuals may recognize that intrapersonal moral hypocrisy is relatively easy to detect, thereby becoming less inclined to act as moral hypocrites.

### 5.4. Limitations

Although this study compared the effects of different intuitive processing modes on moral hypocrisy, as well as the joint effects of negative moods and intuitive processing modes, several limitations should be noted.

First, the sample consisted primarily of Chinese undergraduate students, which represents a relatively restricted population. Therefore, caution should be applied when generalizing the findings to other groups.

Second, this study did not investigate the specific psychological mechanisms through which negative moods and intuitive processing modes influence moral hypocrisy. The explanations provided are primarily inferential, based on theoretical perspectives from previous research. Future studies should further explore the underlying psychological mechanisms. Additionally, how positive emotions and other distinct emotional categories affect moral hypocrisy remains unclear.

Third, using the task assignment paradigm as a measure of intrapersonal moral hypocrisy has certain limitations. The statistical methods applicable to this paradigm are limited (primarily non-parametric tests), and it cannot detect an individual’s “degree of moral hypocrisy”. Employing a within-subjects design for moral judgment tasks also entails certain limitations. These may include the potential for participants to discern the experimental purpose, order effects, and the risk of interference between experimental conditions.

Fourth, this study had a relatively small sample size but observed large effect sizes. This could be influenced by sampling errors and outliers, but it might also reflect a genuinely strong effect.

### 5.5. Future Research Directions

First, different cultural backgrounds (e.g., collectivistic vs. individualistic cultures) may influence individuals’ perceptions of moral hypocrisy. Research has found that individuals in independent cultures tend to hold stricter attitudes toward high-status individuals demonstrating inconsistency between their words and actions (i.e., intrapersonal moral hypocrisy), compared to those in interdependent cultures (Dong et al., 2022). Consequently, future research should further investigate how negative moods and intuitive processing modes influence moral hypocrisy across different cultural contexts. Additionally, it would be essential to diversify sample populations, increase sample sizes and conduct preregistered replication studies.

Second, the use of the task assignment paradigm as a measure of intrapersonal moral hypocrisy presents certain limitations. Future research could explore novel paradigms for measuring intrapersonal moral hypocrisy. For instance, a study has adopted a donation task, where the differences between self-reported donation proportions (the percentage of donated amount relative to total funds) and actual donation proportions serve as the indicator for intrapersonal moral hypocrisy (Fu et al., 2015). Additionally, the disparity of donation amounts in a donation task could serve as an alternative measure for assessing intrapersonal moral hypocrisy. For instance, participants may first be asked to make hypothetical donations using tokens, then be provided with real currency and requested to make an actual donation (Vranka & Houdek, 2024).

Third, this study adopted a within-subjects design for the moral judgment task to assess interpersonal moral hypocrisy (Yu & Zhang, 2024). Other researchers have also employed a within-subjects design in donation tasks to evaluate interpersonal moral hypocrisy. For instance, participants are first asked how much they would be willing to donate, followed by how much they believe others should donate, with the discrepancy between these amounts indicating moral hypocrisy (Polman & Ruttan, 2012). The within-subjects design offers several advantages: calculating the difference in participants’ acceptability of moral violations for different scenarios (self vs. other) can directly reveal the degree of interpersonal moral hypocrisy (Lammers et al., 2010; Lammers, 2012); having the same participant evaluate different scenarios directly reduces error caused by individual differences; and it facilitates the investigation of the combined effects of multiple factors on moral hypocrisy. Future research should examine the reliability of within-subjects moral judgment tasks as a measure of moral hypocrisy.

Fourth, this study examined the relationship between negative moods and moral hypocrisy but did not address the effects of positive moods or different types of emotions on moral hypocrisy. Traditional dual-process models struggle to fully explain the psychological mechanisms through which negative moods increase moral hypocrisy. According to the dual-process 2.0 model, intuitive processing (or System 1) likely comprises distinct types of intuition that compete with each other and vary in their activation strength (Bago & De Neys, 2019; Bago et al., 2021; Capraro, 2024; De Neys, 2023; De Neys & Pennycook, 2019). This perspective partially explains the shared influence of negative moods and cognitive load (or ego depletion) on moral hypocrisy. Future research should continue to investigate the impact of positive moods on moral hypocrisy, as well as the combined effects of emotions and cognitive load (or ego depletion). Furthermore, even emotions with the same valence can induce different cognitive tendencies in individuals (Helion & Ochsner, 2018). Negative moods encompass distinct types such as guilt, anger, fear, and sadness (Gangemi et al., 2025; L. Zhang et al., 2017). For instance, some studies have found that guilt reduces moral hypocrisy (Du et al., 2019), whereas anger increases it (Polman & Ruttan, 2012). Future research needs to examine how different types of positive moods influence moral hypocrisy.

## 6. Conclusions

Intuitive processing modes and negative moods are significant influencing factors of moral hypocrisy, with negative moods exerting a stronger effect. In the neutral mood condition, cognitive load reduced interpersonal and intrapersonal moral hypocrisy; ego depletion significantly increased interpersonal moral hypocrisy and marginally increased intrapersonal moral hypocrisy (*p* = 0.053). In the negative mood condition, cognitive load and ego depletion significantly increased interpersonal moral hypocrisy; however, neither intuitive processing mode significantly increased intrapersonal moral hypocrisy.

## Figures and Tables

**Table 1:** Number and proportion of moral hypocrites under different conditions, n (%), Study 3.

Consistency of Task Choices	Negative Moods			Neutral Moods		
	Control	Cognitive Load	Ego Depletion	Control	Cognitive Load	Ego Depletion
Inconsistency	20 (0.800)	19 (0.826)	23 (0.793)	12 (0.480)	3 (0.107)	20 (0.741)
Consistency	5 (0.200)	4 (0.174)	6 (0.207)	13 (0.520)	25 (0.893)	7 (0.259)
Total	25	23	29	25	28	27

## Data Availability

All data can be accessed at https://osf.io/pve3h/?view_only=47f72701c1f74c11a1e2e5d648c69bdd (accessed on 10 October 2025).

## Appendix A

**Table A1:** Ego depletion manipulation check, Study 1.

	Group	*M*	*SD*	*t*	*df*	*p*	Cohen’s *d*
Task difficulty	Control	2.180	1.137	−8.091	98	<0.001	−1.618
	Ego depletion	4.340	1.507				
Effort expenditure	Control	2.640	1.549	−10.451	98	<0.001	−2.090
	Ego depletion	5.700	1.374				
Fatigue	Control	2.140	1.262	−8.871	98	<0.001	−1.774
	Ego depletion	4.560	1.459				
Depletion	Control	2.340	1.437	−6.424	98	<0.001	−1.285
	Ego depletion	4.260	1.549				

**Table A2:** Ego depletion manipulation check, Study 2.

	Group	*M*	*SD*	*t*	*df*	*p*	Cohen’s *d*
Task difficulty	Control	2.986	1.819	−2.922	137	0.004	−0.496
	Ego depletion	3.857	1.696				
Effort expenditure	Control	3.681	2.125	−5.027	137	<0.001	−0.853
	Ego depletion	5.300	1.645				
Fatigue	Control	2.406	1.556	−8.618	137	<0.001	−1.462
	Ego depletion	4.857	1.788				
Depletion	Control	2.870	1.635	−5.655	137	<0.001	−0.959
	Ego depletion	4.400	1.555				

**Table A3:** A 2 (negative mood vs. neutral mood) × 2 (pre-test vs. post-test) repeated measures ANOVA, Study 2.

	Sum of Squares	*df*	Mean Square	*F*	*p*	$\eta_{p}^{2}$
Within subjects effects						
Time point	4867.618	1	4867.618	478.719	<0.001	0.699
Time point × Mood type	1757.272	1	1757.272	172.824	<0.001	0.456
Residuals	2094.611	206	10.168			
Between subjects effects						
Mood type	4131.541	1	4131.541	58.960	<0.001	0.223
Residuals	14,435.091	206	70.073			

**Table A4:** Multiple comparisons after repeated measures ANOVA, Study 2.

		*M*	*SD*	*t*	*df*	*p*	Cohen’s *d*
Mood type							
Negative mood	Pre-test	9.567	6.849	−24.767	206	<0.001	−1.729
	Post-test	20.519	6.015				
Neutral mood	Pre-test	7.375	5.543	−6.175	206	<0.001	−0.431
	Post-test	10.106	6.832				
Time point							
Pre-test	Negative mood	9.567	6.849	2.537	206	0.012	0.346
	Neutral mood	7.375	5.543				
Post-test	Negative mood	20.519	6.015	11.667	206	<0.001	1.644
	Neutral mood	10.106	6.832				

**Table A5:** Ego depletion manipulation check, Study 3.

	Group	*M*	*SD*	*t*	*df*	*p*	Cohen’s *d*
Task difficulty	Control	2.457	1.125	−8.237	139	<0.001	−1.387
	Ego depletion	4.225	1.406				
Effort expenditure	Control	3.071	1.255	−8.997	139	<0.001	−1.515
	Ego depletion	5.141	1.467				
Fatigue	Control	2.429	1.071	−12.459	139	<0.001	−2.099
	Ego depletion	5.085	1.432				
Depletion	Control	2.643	1.252	−9.216	139	<0.001	−1.552
	Ego depletion	4.549	1.205				

**Table A6:** A 2 (negative mood vs. neutral mood) × 2 (pre-test vs. post-test) repeated measures ANOVA, Study 3.

	Sum of Squares	*df*	Mean Square	*F*	*p*	$\eta_{p}^{2}$
Within subjects effects						
Time point	2021.261	1	2021.261	216.106	<0.001	0.508
Time point × Mood type	214.152	1	214.152	22.896	<0.001	0.099
Residuals	1954.801	209	9.353			
Between subjects effects						
Mood type	58.860	1	58.860	3.204	0.075	0.015
Residuals	3839.420	209	18.370			

**Table A7:** Multiple comparisons after repeated measures ANOVA, Study 3.

		*M*	*SD*	*t*	*df*	*p*	Cohen’s *d*
Mood type							
Negative mood	Pre-test	7.170	2.535	−13.811	209	<0.001	−1.558
	Post-test	12.972	4.086				
Neutral mood	Pre-test	7.848	2.752	−6.995	209	<0.001	−0.793
	Post-test	10.800	4.980				
Time point							
Pre-test	Negative mood	7.170	2.535	−1.861	209	0.064	−0.182
	Neutral mood	7.848	2.752				
Post-test	Negative mood	12.972	4.086	3.465	209	<0.001	3.465
	Neutral mood	10.800	4.980				
